# Association of the PCSK6 rs1531817(C/A) polymorphism with the prognosis and coronary stenosis in premature myocardial infarction patients: a prospective cohort study

**DOI:** 10.1186/s12944-024-02206-w

**Published:** 2024-07-22

**Authors:** Li Sun, Jing-xian Wang, Jing Ma, Xu Zhang, Yu-Hang Wang, An-Ran Jing, Miao-Miao Liang, Jing-yu Liu, Yin Liu, Jing Gao

**Affiliations:** 1 Graduate School, Tianjin Medical University, No.22 Qi Xiang Tai Road, Tianjin, 300070 Heping District P.R. China; 2 Department of Cardiology, Zoucheng Peoples Hospital, No. 59 Qianquan Road, Zoucheng, 273500 Shandong P.R. China; 3 Thoracic Clinical College, Tianjin Medical University, No.22 Qi Xiang Tai Road, Tianjin, 300070 Heping District P.R. China; 4 Department of Cardiology, Tianjin Chest Hospital, No.261 Tai Erzhuang Road, Tianjin, 300222 Jinnan District P.R. China; 5 Cardiovascular Institute, Tianjin Chest Hospital, No.261 Tai Erzhuang Road, Tianjin, 300222 Jinnan District P.R. China; 6 Tianjin Key Laboratory of Cardiovascular Emergency and Critical Care, Tianjin, P.R. China; 7 Chest Hospital, Tianjin University, No.92 Weijin Road, Tianjin, 300072 Nankai District P.R. China

**Keywords:** Premature myocardial infarction, Proprotein convertase subtilisins/Kexin 6, Single nucleotide polymorphism, Coronary stenosis, Major adverse cardiovascular events

## Abstract

**Background:**

Proprotein convertase subtilisins/kexin 6 (PCSK6) polymorphisms have been shown to be associated with atherosclerosis progression. This research aimed to evaluate the relationship of PCSK6 rs1531817 polymorphisms with coronary stenosis and the prognosis in premature myocardial infarction (PMI) patients.

**Methods:**

This prospective cohort analysis consecutively included 605 PMI patients who performed emergency percutaneous coronary intervention (PCI) at Tianjin Chest Hospital sequentially between January 2017 and August 2022, with major adverse cardiovascular events (MACEs) as the outcome. Analyses assessed the relationships among PCSK6 rs1531817 polymorphism, Gensini score (GS), triple vessel disease (TVD), and MACEs.

**Results:**

92 (16.8%) patients experienced MACEs with an average follow-up of 25.7 months. Logistic analysis revealed that the PCSK6 rs1531817 CA + AA genotype was an independent protective factor against high GS and TVD. Cox analysis revealed that the PCSK6 rs1531817 CA + AA genotype was an independent protective factor against MACEs. The mediation effect results showed that apolipoprotein A1/apolipoprotein B (ApoA1/ApoB) partially mediated the association between PCSK6 rs1531817 polymorphism and coronary stenosis and that total cholesterol/high-density lipoprotein (TC/HDL) and TVD partially and in parallel mediated the association between the PCSK6 rs1531817 polymorphism and MACEs.

**Conclusion:**

Patients with the PCSK6 CA + AA genotype have milder coronary stenosis and a better long-term prognosis; according to the mediation model, ApoA1/ApoB and TC/HDL partially mediate. These results may provide a new perspective on clinical therapeutic strategy for anti-atherosclerosis and improved prognosis in PMI patients.

**Supplementary Information:**

The online version contains supplementary material available at 10.1186/s12944-024-02206-w.

## Introduction

In recent years, an increasing proportion of younger patients are being hospitalized for acute myocardial infarction (AMI) [1–3]. Premature myocardial infarction (PMI), with its high morbidity and mortality, is a significant problem for the health of the general population [4, 5]. This research team has previously conducted a series of long-term studies on coronary stenosis and prognostic risk in PMI patients from a clinical perspective [6, 7]. Given that PMI has 63% heritability, genetics is essential for its development [8]. Therefore, it is important to explore the factors affecting coronary stenosis and prognosis in PMI patients from a genetic perspective for clinical management.

Proprotein convertase subtilisins/kexin 6 (PCSK6) is expressed in liver cells, and its encoding gene is located at q26.3 on the 15th chromosome [9]. It regulates protein structure and function through protein hydrolysis cleavage. Some studies suggest that PCSK6 is associated with atherosclerosis [10, 11]. In a recent study, 3378 individuals with high coronary heart disease risk to investigate the relationship between PCSK6 polymorphisms and the progression of carotid intima-media thickness (cIMT). The results showed that mutations in the PCSK6 rs1531817 (C > A) were significantly associated with maximal progression of internal carotid artery thickness; the patients carrying the A allele showed reduced cIMT progression compared to individuals with CC genotypes (from the AtheroExpress Biospecimen Library) [10]. cIMT is a predictive surrogate for atherosclerosis, suggesting that atherosclerotic disease progression is related to the PCSK6 rs1531817 mutation.

A previous study has shown that PCSK6 polymorphisms are associated with atherosclerosis progression [10]. Cardiac fibroblasts and endothelial cells have been demonstrated to express PCSK6 [12]. However, it is still unknown whether the PCSK6 rs1531817 polymorphism is associated with coronary stenosis and the prognosis in PMI patients. There are no relevant studies available. Therefore, the study aimed to evaluate the relationship between PCSK6 rs1531817 polymorphism, coronary stenosis, and the prognosis in PMI patients.

## Methods

### Study population

This prospective cohort analysis consecutively included 671 PMI patients who performed emergency percutaneous coronary intervention (PCI) at Tianjin Chest Hospital sequentially between January 2017 and August 2022. There is no standardized age limit for PMI. Current national and international studies generally set the upper age limit for PMI at 45–55 years [13–16]. This study defined PMI as ≤ 50 years for men and ≤ 55 years for women. The inclusion criteria were (1) meeting age inclusion criteria. (2) Fulfilment of AMI diagnosis. (3) Complete coronary angiography and underwent PCI. (4) Informed permission was signed by each patient who was included. The exclusion criteria were (1) severe liver or kidney disease. (2) Having infections or rheumatologic or immunologic diseases. (3) Patients with malignant tumours requiring treatment. (4) Missing data related to PCSK6 rs1531817 genotypes and coronary angiography. This study used the fourth international definition of MI (2018) to diagnose AMI [17]: clinical proof of acute myocardial ischaemia coupled with damage; elevated or reduced cTnT (at least one time above the top 99% threshold), as well as any one of these subsequent circumstances (1) symptoms and signs of myocardial ischaemia; (2) novel ischaemic electrocardiographic alterations; (3) patient characteristics of the Q-wave; (4) imaging evidence indicating that new focal wall motion abnormalities or novel patterns of myocardial inactivation are compatible with pathologic alterations of ischaemia; or (5) coronary thrombosis discovered by autopsy or radiography. After the screening, 605 patients were ultimately included.

**Fig. 1:**
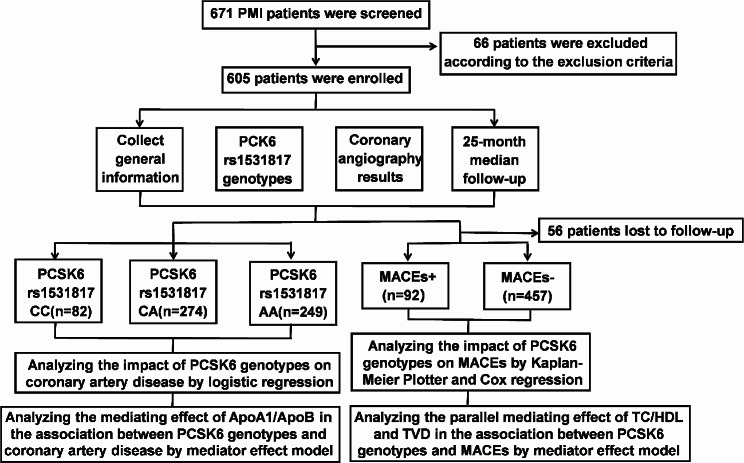
Cohort study flowchart

The Tianjin Chest Hospital’s Ethical Commission approved the study (No.2017KY-007-01), and this study received informed consent from each patient. Figure 1 displays the flowchart for the cohort study.

### General data collection

Age, sex, medical history, admission blood pressure, heart rate were all recorded as each patient’s clinical baseline data. The patients’ laboratory examination data, echocardiography, and other relevant indicators were also collected. According to the normal reference range of laboratory indicators in Tianjin Chest Hospital, patients with one or more abnormalities of the following indicators were considered dyslipidemia: total cholesterol (TC); triglycerides (TG); low density lipoprotein (LDL); and high density lipoprotein (HDL).

### Coronary angiography and intervention

Two cardiologists qualified to diagnose and treat coronary arteries performed coronary angiography and PCI. The GS was computed for each enrolled PMI patient based on the results of their coronary angiography. Each coronary artery stenosis was ranked according to a score of 1 for 1–25%, 2 for 26–50%, 4 for 51–75%, 8 for 76–90%, 16 for 91–99%, and complete arterial occlusion 32. After that, the score was multiplied with a factor. The multiplicand is as follows: the coefficient of multiplication was five for left main cardiac artery, two and a half for left anterior descending and circumflex arteries’ proximities, one and a half for medial left anterior descending branch. The coefficient of multiplication was one for right coronary artery, circumflex’s median and distant segment, and distant segment of left anterior descending; with every other segment, the multiplication factor was 0.5. The total coronary artery score is taken as the GS [18–20].

The present study used MACEs and time-to-MACEs for implicit variables. GSs were stratified into three levels using X-tile software: a low-GS group (< 52.0 points, *n* = 323), a medium-GS group (52.0–94.0 points, *n* = 215), and a high-GS group (> 94.0 points, *n* = 67), which represented mild, moderate, and severe coronary stenosis, respectively. Table 1 shows the estimated cut-off point range of the value region as well as the distributional features of the GS levels.

**Table 1:** Cut-off point of GS points by X-tile method

Group	Range(points)	Patients	Percentage(%)	MACEs(%)
Low GS	5–52	286	52.09	40(13.99)
Medium GS	52–94	199	36.25	33(16.58)
High GS	94–202	64	11.66	19(29.69)
Total	5−202	549	100.00	92(16.76)

*GS* Gensini score; *MACEs* major adverse cardiovascular events

Patients were categorized according to the number of branches involved (≥ 50% stenosis in the major coronary artery) in their coronary lesions into groups of single lesions, double lesions, and triple lesions. Double vessel disease (DVD) was considered when stenosis of the left main lesion involved, regardless of the involvement of the left anterior descending, circumflex, or both; triple vessel disease (TVD) was considered when accompanied by a right coronary arterial lesion.

### Genetic analysis

Blood samples were collected from every individual after fasting on the first day after admission. Ten minutes were spent spinning the blood samples at four degrees Celsius and three thousand rotations per minute; moreover, the serum was kept at -80 degrees Celsius in the Chest Hospital Biospecimen Bank. Genomic DNA was isolated and purified from 1 mL EDTA-treated peripheral blood by a blood DNA extraction kit (LaServ, Shanghai, China) and subjected to quality control. For the purpose of testing single nucleotide polymorphism (SNP) sites, MassARRAY Assay Design 3.1 software and genotyping tools from Sequenom were utilized to construct single-base extension primers and polymerase chain reaction (PCR) amplification primers, and the designed primers were submitted to a company for synthesis. The primers were centrifuged under vacuum, and then water was added. After adding water, shaking, and mixing, the primers were diluted with 36 µL water per 1 OD and left at room temperature for thirty min. Next, PCR amplification was carried out, and the PCR amplification system consisted of water (1.8 µl), 10× PCR buffer (0.5 µl), PCR primer combination (1 µl), MgCl2 (0.4 µl), dNTP mixture (0.1 µl), HotStar Taq (5 U/µl) (0.2 µl), and DNA sample (1 µl). The cycling program consisted of 120 s at 95 °C, 45 cycles of 30 s at 56 °C, and 180 s at 72 °C. Subsequently, the PCR products were treated with alkaline phosphatase and subjected to a single-base extension reaction. The PCSK6 rs1531817 genotyping analysis of the included population was performed using the nucleic acid mass spectrometry analysis system of the MassARRAY platform of Beijing COMPASS BIOTECHNOLOGY Co., Ltd. (Sequenom, San Diego, CA, USA) after resin purification and microarray spotting.

### Follow-up

Trained nurses or cardiologists monitored every patient involved in the trial for at least 12 months through phone follow-ups, outpatient check-ups, and clinic visits. The follow-up endpoint was defined as the occurrence of a major adverse cardiovascular event (MACE). MACEs consisted of cardiac death, nonfatal stroke, readmission for ischaemic cardiovascular events, target lesion revascularization (TLR), and rehospitalization for cardiac insufficiency. Ischaemic cardiovascular events included acute myocardial infarction and episodes of unstable angina.

### Statistical methods

Using the X-tile technique, the GS was split into three categories: low, medium, and high. The cut-off points were selected via an X-tile analysis, which considered covariates and patient survival, to maintain homogeneity and variability within the groups [21].

Categorical variables are stated in numerical and percentage form. All of the continuous variables in this study were nonnormally distributed, and they are expressed as medians and quartiles. Chi-squared tests were applied to evaluate the differences among the categories of categorical data, while nonparametric tests were used to assess the differences among the categories of continuous data. The effect of the PCSK6 rs1531817 genotype on coronary stenosis was examined by logistic regression. The associations between the PCSK6 rs1531817 genotype and MACEs were analyzed by Cox regression and Kaplan‒Meier curves [22]. The mediating effect of lipids associated with the PCSK6 rs1531817 polymorphism, coronary stenosis, and MACEs was investigated via a mediating effect model. The mediating effect analysis was performed using the bootstrap test procedure, and the inverse logarithmic transformation was used to convert the mediating effect values to the corresponding odds ratio.

The data analysis program SPSS 26.0 was used for this research, GraphPad Prism 8.0 was used for all graphs, X-tile analyses were conducted with the X-tile system (Copyright Yale University), and the mediation effects model was analyzed by MPLUS 8.3. *P* < 0.05 were employed to identify statistically significant differences.

## Results

### Basic characteristics of various PCSK6 rs1531817 genotypes

**Fig. 2:**
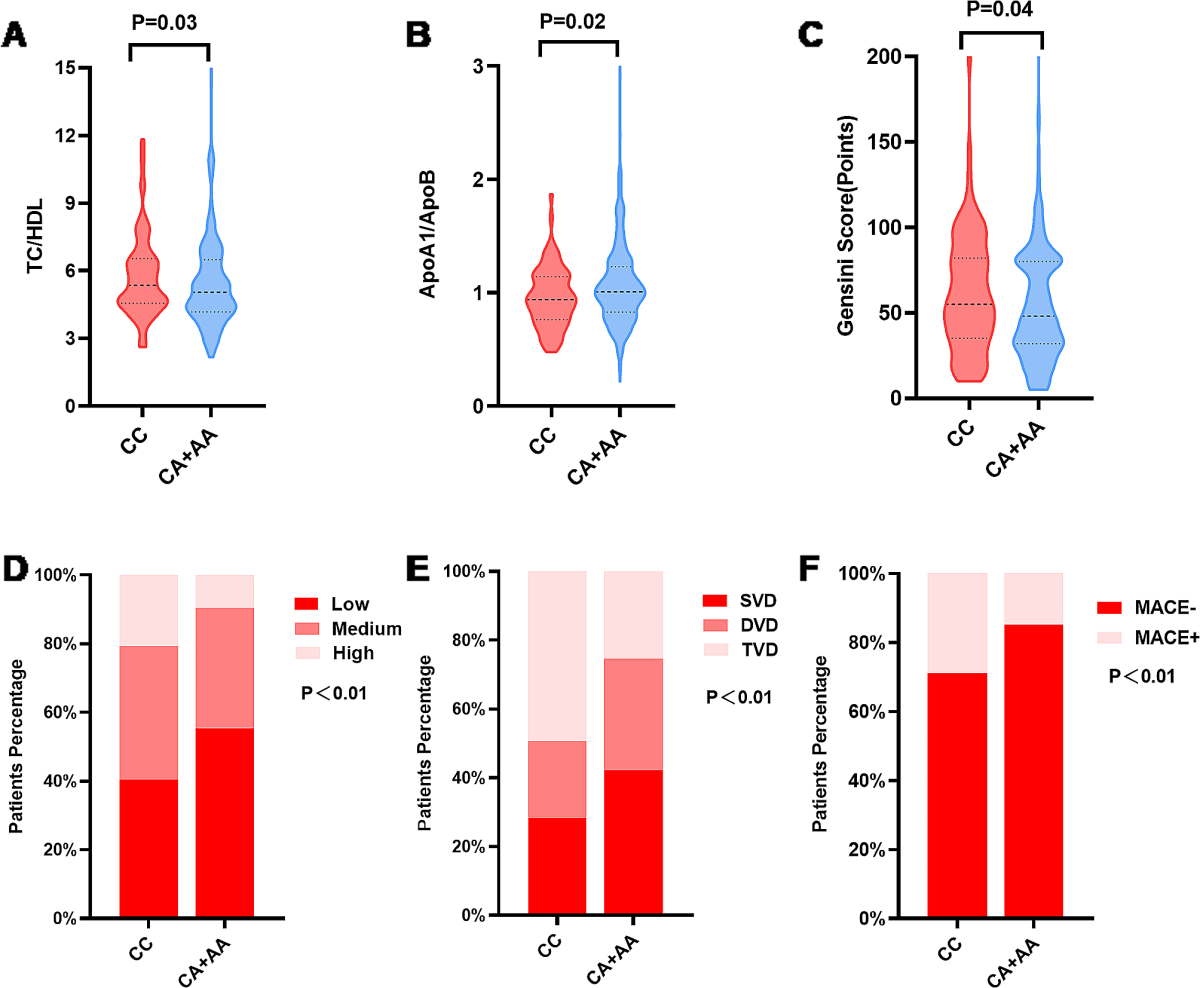
Baseline data were compared between the PCSK6 rs1531817 CC and CA + AA genotypes. **A-C** Comparison of TC/HDL, ApoA1/ApoB, and GS levels between the PCSK6 rs1531817 CC and CA + AA genotypes. **D-F** The percentage of patients with different GS subgroups, percentage of patients with different coronary lesions, and incidence of MACEs were compared between the two groups

When comparing the PCSK6 rs1531817 genotypes, differences in TC/HDL, ApoA1/ApoB, GS group, number of branches involved in coronary lesions, MACEs, and TLR were statistically significant (*P* < 0.05). CA + AA genotypes patients had considerably lower TC/HDL and Gensini scores and significantly greater ApoA1/ApoB levels than CC genotype patients. Fewer patients with the PCSK6 rs1531817 CA + AA genotype had a high GS, TVD, and MACEs than patients with the CC genotype (*P* < 0.05) (Table 2; Fig. 2).

**Table 2:** Baseline characteristics of various PCSK6 rs1531817 genotypes

Characteristics	Wild type CC(*n* = 82)	Heterozygous type CA(*n* = 274)	Mutant type AA(*n* = 249)	*P* value
Male, n(%)	74(90.24)	243(88.69)	220(88.35)	0.89
Age, years	42.00(37.00,44.25)	42.00(38.00,45.00)	42.00(37.50,45.50)	0.58
BMI, kg/m2	26.12(24.21,28.69)	26.12(24.09,28.41)	25.83(23.71,28.37)	0.59
History, n(%)				
Smoking	51(62.20)	184(67.15)	164(65.86)	0.71
Alcohol intake	27(32.93)	103(37.59)	78(31.33)	0.31
Hypertension	33(40.24)	136(49.64)	110(44.18)	0.24
Diabetes	14(17.07)	45(16.42)	51(20.48)	0.47
Dyslipidemia	71(86.58)	223(81.98)	204(81.93)	0.59
Previous Stroke	1(1.22)	7(2.55)	11(4.42)	0.25
STEMI, n(%)	67(81.71)	234(85.40)	214(85.94)	0.64
Systolic pressure, mmHg	132.00 (125.00,148.00)	134.00 (120.00,145.00)	132.00 (120.00,145.00)	0.46
Diastolic pressure, mmHg	83.50(72.00,95.25)	80.00(70.00,91.25)	80.00(70.00,92.00)	0.47
Heart rate, bpm	78.00(70.00,90.00)	77.00(68.75,88.25)	76.00(69.00,86.00)	0.36
Biochemical characteristics				
WBC,10^9/L	10.26(8.44,12.10)	10.59(8.94,12.70)	10.99(9.06,13.14)	0.12
CRP, mg/L	4.93(2.04,10.98)	5.50(2.44,12.39)	5.61(2.23,12.23)	0.84
ALT, U/L	44.75(30.18,73.23)	46.35(30.60,71.38)	46.50(32.15,69.60)	0.99
Cr, umol/L	76.00(68.75,84.50)	73.00(63.75,81.00)	74.00(65.00,84.00)	0.11
FBG, mmol/L	5.85(5.11,7.29)	6.06(5.30,7.78)	5.83(5.18,8.69)	0.71
TC, mmol/L	4.87(4.28,5.58)	4.79(4.12,5.42)	4.76(4.11,5.58)	0.61
TG, mmol/L	2.10(1.56,3.09)	1.93(1.42,2.80)	2.11(1.48,3.01)	0.18
HDL, mmol/L	0.92(0.80,1.01)	0.95(0.82,1.13)	0.92(0.80,1.06)	0.05
LDL, mmol/L	3.31(2.71,3.76)	3.22(2.58,3.81)	3.16(2.50,3.86)	0.66
TC/HDL	5.36(4.57,6.55)	4.92(4.18,5.94)	5.25(4.17,6.35)	0.02
ApoA1,g/L	1.09(0.97,1.23)	1.14(1.02,1.29)	1.13(1.01,1.26)	0.20
ApoB, g/L	1.17(0.99,1.34)	1.14(0.92,1.30)	1.13(0.93,1.35)	0.36
ApoA1/ApoB	0.94(0.76,1.14)	1.01(0.87,1.22)	1.01(0.79,1.24)	0.04
cTnT, ng/ml	2.44(1.19,5.17)	2.74(1.06,5.67)	3.14(1.40,5.43)	0.34
BNP, pg/ml	213.08 (69.30,524.50)	234.46 (77.12,671.00)	267.70 (75.92,839.00)	0.43
D-Dimer, ug/ml	0.28(0.21,0.44)	0.29(0.22,0.47)	0.31(0.22,0.54)	0.42
Fg, g/L	3.33(2.88,3.86)	3.35(2.88,3.92)	3.23(2.84,3.82)	0.52
Echocardiography				
LVEF,%	53.00(47.00,56.00)	52.00(46.00,57.00)	51.00(45.00,56.00)	0.41
PAP, mmHg	30.00(30.00,30.00)	30.00(30.00,30.00)	30.00(30.00,30.00)	0.84
Gensini Score, points	55.00(35.00,82.00)	48.00(31.50,80.00)	46.00(32.00,80.00)	0.12
Gensini Score Group, n(%)				<0.01
<52	33(40.24)	149(54.38)	141(56.63)	
52–94	32(39.02)	103(37.59)	80(32.13)	
>94	17(20.73)	22(8.03)	28(11.24)	
Coronary vessel disease, n(%)				<0.01
SVD	24(29.27)	116(42.34)	111(44.58)	
DVD	18(21.95)	90(32.8)	76(30.52)	
TVD	40(48.78)	68(24.82)	62(24.90)	
Medication during follow-up, n(%)				
DAPT	81(98.78)	274(100.0)	247(99.20)	0.26
Statin	81(98.78)	270(98.54)	244(97.99)	0.84
Anticoagulant	74(90.24)	246(89.78)	227(91.16)	0.86
ACEI/ARB	67(81.71)	214(78.10)	199(79.92)	0.75
Beta-blocker	71(86.59)	241(87.96)	210(84.34)	0.48
MACEs, n(%)	22(26.83)	37(13.50)	33(13.25)	<0.01
Cardic death	2(2.44)	2(0.73)	1(0.40)	0.22
Non-fatal stroke	1(1.22)	3(1.09)	0(0.0)	0.26
Hospital admission for Ischemic cardiovascular events	13(15.85)	22(8.03)	20(8.03)	0.09
Target lesion revascularization	10(12.20)	7(2.55)	10(4.02)	<0.01
Hospital admission for HF	3(3.66)	11(4.01)	11(4.42)	0.91

*PCSK6* proprotein convertase subtilisin/kexin type 6; *STEMI* ST-segment elevation myocardial infarction; *BMI* body mass index; *WBC* white blood cell; *ALT* alanine transaminase; *CRP* C-reactive protein; *Cr* creatinine; *FBG* fasting blood glucose; *TC* total cholesterol; *TG* Triglyceride; *HDL* high-density lipoprotein; *LDL* low-density lipoprotein; *Apo* apolipoprotein; *cTnT* cardiac troponin T; *BNP* B type natriuretic peptide; *Fg* Fibrinogen; *LVEF* left ventricular ejection fraction; *PAP* pulmonary artery pressure; *SVD s*ingle vessel disease; *DVD* double vessel diseas; *TVD* triple vessel diseases; *DAPT* dual antiplatelet therapy; *ACEI* angiotensin-converting enzyme inhibitors; *ARB* angiotensin II receptor blockers; *MACEs* major adverse cardiovascular events

Data are present as mean (inter-quartile range) or number (%)

### Correlation of the PCSK6 rs1531817 polymorphism with the Gensini score in PMI patients

Among the various GS groups, hypertension, diabetes, previous stroke, heart rate, white blood cell (WBC), fasting blood glucose (FBG), alanine transaminase (ALT), B type natriuretic peptide (BNP), TC, LDL, ApoB, ApoA1/ApoB, cardiac troponin T (cTnT), fibrinogen (Fg), and PCSK6 rs1531817 genotype (CA + AA vs. CC) were significant differences. Compared to the low and medium GS groups, the percentage of patients with CA + AA genotypes was considerably lower in the high GS group (*P* < 0.05) (Table S1).

Univariate logistic analysis included indicators with *P* < 0.05 for comparisons between different GS groups. TC, LDL, and ApoB were not included in the logistic regression since ApoA1/ApoB was significantly correlated with TC (*r*=-0.62, *P* < 0.01), LDL (*r*=-0.65, *P* < 0.01), and ApoB (*r*=-0.79, *P* < 0.01). Univariate logistic regression revealed that diabetes, previous stroke, heart rate, WBC, ALT, FBG, cTnT, and Fg were risk factors for the high GS, ApoA1/ApoB, and PCSK6 rs1531817 CA + AA genotypes were protective factors against the occurrence of high GS (Fig. S1 A). The multivariate logistic regression considered the indicators from the univariate logistic regression with *P* < 0.05, compared to the low GS, FBG, cTnT, and Fg were risk factors for the high GS. The CA, AA, and CA + AA genotypes were the protective factors (adj*OR* = 0.30, 95% CI: 0.14–0.63, *P* < 0.01) (Fig. S1 B, D). Compared to the medium GS group, the risk factor for the high GS group was previous stroke, and the protective factors were the CA and CA + AA genotypes (adj*OR* = 0.45, 95% CI: 0.21–0.96, *P* = 0.04) (Fig.S1 C, E).

### Correlation of PCSK6 rs1531817 polymorphisms with the number of branches involved in coronary stenosis in individuals with PMI

Age, sex, hypertension status, diabetes, ALT, FBG, TC, LDL, TC/HDL, ApoB, ApoA1/ApoB, cTnT, Fg, and PCSK6rs1531817 genotypes were the variables tested in the comparison among patients with single vessel disease (SVD), DVD, and TVD. Among them, compared to the SVD and DVD groups, the percentage of patients with CA + AA genotypes was decreased in the TVD group (*P* < 0.05) (Table S2).

**Fig. 3:**
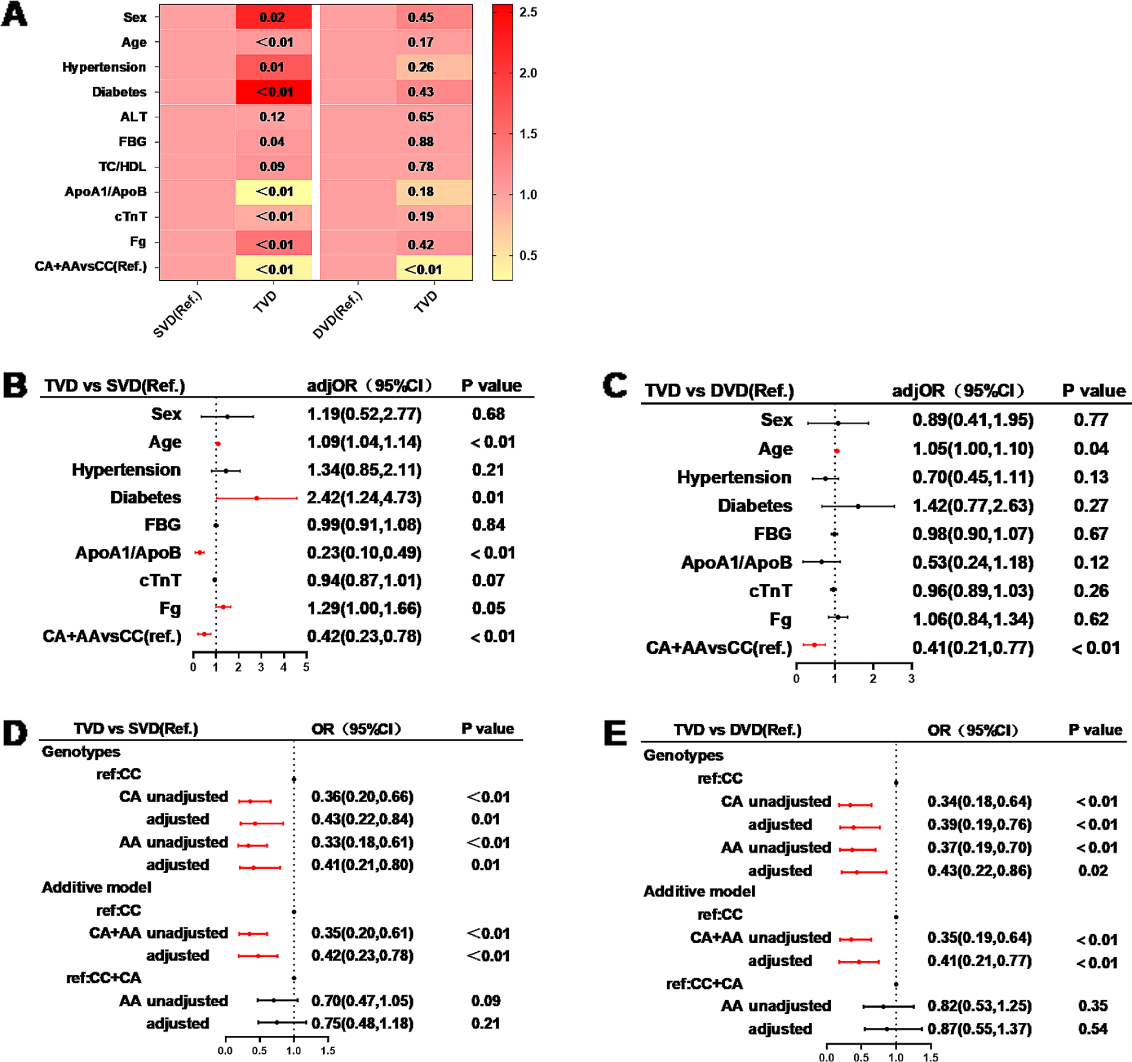
Correlation between the number of coronary stenosis branches and PCSK6 rs1531817 polymorphisms in PMI patients. **A** Heatmap showing the ORs and P values for the risk of TVD according to univariate logistic regression (red and yellow indicate positive and negative correlations, respectively). **B**,** C** Multivariate logistic regression for the risk of TVD. **D**,** E** Logistic regression of different genotypes of PCSK6 rs1531817 for the risk of TVD (TVD vs. SVD, TVD vs. DVD). Multivariate logistic regression was adjusted for FBG, Fg, ApoA1/ApoB, cTnT, age, sex, hypertension, and diabetes. *OR* odds ratio; *CI* confidence intervals

The univariate logistic analysis included indicators with *p* < 0.05 for comparisons among the SVD, DVD, and TVD patients. TC, LDL, and ApoB were not included in the logistic regression since ApoA1/ApoB was significantly related to TC, LDL, and ApoB. Univariate logistic regression revealed that age, female sex, hypertension status, diabetes status, FBG, and Fg were risk factors for TVD. The ApoA1/ApoB, cTnT, and PCSK6 rs1531817 CA + AA genotypes were protective factors for TVD in patients with PMI (*P* < 0.05) (Fig. 3A). The multivariate logistic regression considered the indicators from the univariate logistic regression with *P* < 0.05, which suggested that age and FBG were risk factors for TVD compared with the SVD group, and the protective factors were ApoA1/ApoB and the CA, AA, and CA + AA genotypes (adj*OR* = 0.42, 95% CI: 0.23–0.78, *P* < 0.01) (Fig. 3B, D). Age was a risk factor for TVD, and protective factors were the CA, AA, and CA + AA genotypes (adj*OR* = 0.41, 95% CI: 0.21–0.77, *P* < 0.01) compared to the DVD group (Fig. 3C, E).

### Correlation of the PCSK6 rs1531817 polymorphism with MACEs

The most crucial social genetic component is the Hardy‒Weinberg equilibrium (HWE) [23, 24]; if the population complies with the HWE (*P* > 0.05), the population is from the same Mendelian stock and the samples are representative of population genetic studies. After testing HWE for both the overall patient group and the loss to follow-up, MACEs-positive and MACEs-negative groups, it was found that the distribution of each genotype group of PCSK6 rs1531817 complied with the HWE (*P* > 0.05) (Table S3 and Table 3).

**Table 3:** Analysis of clinical data of PMI patients with MACEs

Characteristics	MACEs(*n* = 92)	Non-MACEs (*n* = 457)	*P* value
Male, n(%)	81(88.04)	408(89.28)	0.73
Age, years	41.00(37.00,45.00)	42.00(38.00,45.00)	0.44
BMI, kg/m2	26.73(24.77,29.41)	25.98(23.77,28.36)	0.02
History, n(%)			
Smoking	63(68.48)	300(65.65)	0.60
Alcohol intake	35(38.04)	155(33.92)	0.45
Hypertension	45(48.91)	206(45.08)	0.50
Diabetes	20(21.74)	77(16.85)	0.26
Previous Stroke	5(5.43)	12(2.63)	0.16
STEMI, n(%)	71(77.17)	394(86.21)	0.03
Systolic pressure, mmHg	135.00(121.25,150.00)	132.00(120.00,145.00)	0.23
Diastolic pressure, mmHg	82.00(73.00,94.75)	80.00(70.00,92.50)	0.81
Heart rate, bpm	80.00(71.00,90.00)	76.00(69.00,86.00)	0.12
Biochemical haracteristics			
WBC,10^9/L	10.74(8.81,13.33)	10.58(8.91,12.68)	0.40
CRP, mg/L	6.23(3.23,10.81)	5.36(2.06,12.87)	0.41
ALT, U/L	43.40(29.05,67.00)	47.40(32.20,72.80)	0.22
Cr, umol/L	75.50(64.00,89.50)	74.00(64.50,82.00)	0.29
FBG, mmol/L	6.29(5.07,9.26)	5.86(5.25,7.97)	0.42
TC, mmol/L	4.94(4.36,5.67)	4.78(4.14,5.44)	0.09
TG, mmol/L	2.11(1.49,3.13)	2.05(1.46,2.81)	0.20
HDL, mmol/L	0.92(0.77,1.06)	0.94(0.81,1.10)	0.12
LDL, mmol/L	3.37(2.70,3.92)	3.20(2.53,3.79)	0.28
TC/HDL	5.42(4.38,6.49)	4.98(4.18,6.15)	0.03
ApoA1,g/L	1.12(1.01,1.26)	1.13(1.00,1.27)	0.90
ApoB, g/L	1.22(1.05,1.38)	1.13(0.93,1.32)	0.05
ApoA1/ApoB	0.94(0.80,1.15)	1.02(0.82,1.22)	0.05
cTnT, ng/ml	2.95(1.28,5.55)	2.90(1.15,5.57)	0.75
BNP, pg/ml	343.47(75.39,909.13)	232.79(78.52,650.00)	0.27
D-Dimer, ug/ml	0.30(0.21,0.57)	0.29(0.22,0.48)	0.96
Fg, g/L	3.44(3.07,3.85)	3.24(2.84,3.84)	0.04
Echocardiography			
LVEF,%	50.00(42.00,55.75)	51.00(46.00,56.00)	0.03
PAP, mmHg	30.00(30.00,30.00)	30.00(30.00,30.00)	<0.01
Gensini Score, points	55.00(32.00,83.75)	48.00(32.00,80.00)	0.14
High GS group, n(%)	19(20.65)	45(9.85)	<0.01
TVD, n(%)	39(42.39)	120(26.26)	<0.01
PCSK6 genotypes			<0.01
CC	22(23.91)	54(11.82)	
CA	37(40.22)	216(47.26)	
AA	33(35.87)	187(40.92)	
Additive model			
Dominant model (AA + CAvsCC)	70(76.09)	403(88.18)	<0.01
Recessive model (AAvsCA + CC)	33(35.87)	187(40.92)	0.37
Medication during follow-up, n(%)			
DAPT	91(98.91)	455(99.56)	0.44
Statin	89(96.74)	451(98.69)	0.18
Anticoagulant	82(89.13)	417(91.25)	0.52
ACEI/ARB	74(80.43)	358(78.34)	0.65
Beta-blocker	80(86.96)	396(86.65)	0.94
HWE X^2	3.11	0.49	
HWE P	0.08	0.48	

*PCSK6* proprotein convertase subtilisin/kexin type 6; *STEMI* ST-segment elevation myocardial infarction; *BMI* body mass index; *WBC* white blood cell; *ALT* alanine transaminase; *CRP* C-reactive protein; *Cr* creatinine; *FBG* fasting blood glucose; *TC* total cholesterol; *TG* Triglyceride; *HDL* high-density lipoprotein; *LDL* low-density lipoprotein; *Apo* apolipoprotein; *cTnT* cardiac troponin T; *BNP* B type natriuretic peptide; *Fg* Fibrinogen; *LVEF* left ventricular ejection fraction; *PAP* pulmonary artery pressure; *TVD* triple vessel diseases; *DAPT* dual antiplatelet therapy; *ACEI* angiotensin-converting enzyme inhibitors; *ARB* angiotensin II receptor blockers; *MACEs* major adverse cardiovascular event; *HWE* Hardy–Weinberg law of equilibrium

Data are present as mean (inter-quartile range) or number (%)

56 patients (9.26%) were lost to follow-up in total. The baseline information of these 56 patients and overall patients were compared, and we discovered that the clinical markers did not differ between the two cohorts (*P*>0.05) (Table S3). Therefore, this study excluded these 56 patients from the association analysis between the PCSK6 rs1531817 genotype and MACEs. The follow-up of 92 (16.8%) patients with MACEs revealed five cardiac deaths (0.9%), four nonfatal strokes (0.7%), 55 readmissions for ischaemic cardiovascular events (10.0%), 27 target lesion revascularization (4.9%) and 25 hospitalizations for heart failure (4.6%). Each clinical index was compared between the MACEs and non-MACEs groups; the two groups differed considerably on the basis of body mass index (BMI), STEMI, TC/HDL, Fg, LVD, LVEF, PAP, high GS, TVD, and PCSK6 rs1531817 genotypes (*P* < 0.05) (Table 3).

**Fig. 4:**
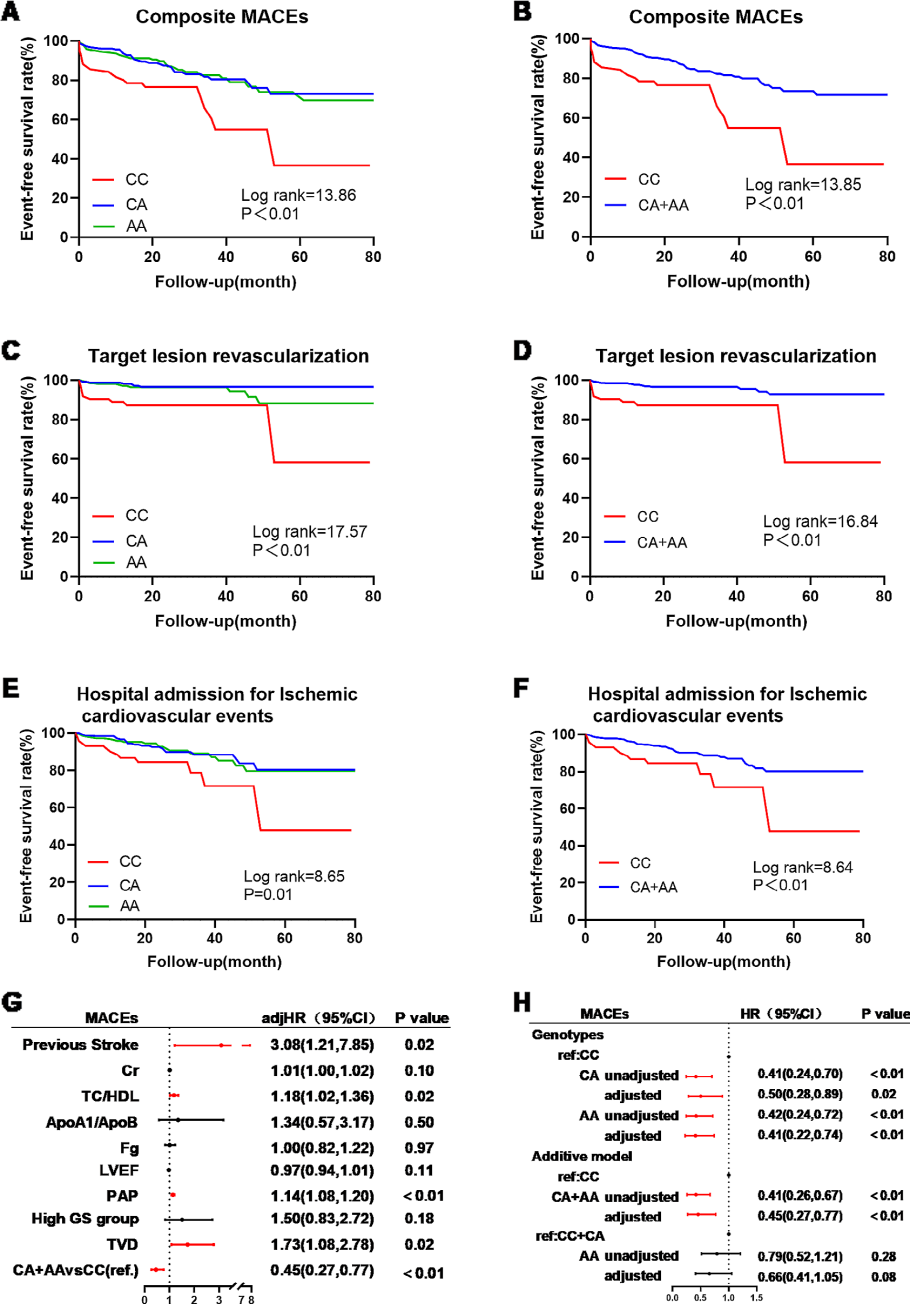
Correlation of the PCSK6 rs1531817 polymorphism with MACEs. **A**,** B** Kaplan–Meier curve for composite MACEs based on the genotype of PCSK6 rs1531817. **C**,** D** Kaplan–Meier curve for target lesion revascularization based on the genotype of PCSK6 rs1531817. **E**,** F** Kaplan–Meier curve for hospital admission for ischaemic cardiovascular events based on the genotype of PCSK6 rs1531817. **G** Multivariate Cox regression analysis of MACEs in PMI patients. **H** Cox regression of the PCSK6 rs1531817 genotype for the occurrence of MACEs. Multivariate Cox regression was adjusted for previous stroke, Cr, TC/HDL, ApoA1/ApoB, Fg, LVEF, PAP, high GS group, and TVD. *HR* hazard ratio; *CI* confidence interval

Kaplan-Meier curve analysis showed that composite MACEs, target lesion revascularization, and readmission for ischaemic cardiovascular events were significantly associated with the PCSK6 rs1531817 genotype. Patients with the CC genotype had shorter event-free survival than those with the CA + AA genotype (*P* < 0.05) (Fig. 4A-F).

Univariate Cox regression revealed that history of stroke, creatinine (Cr), left ventricular ejection fraction(LVEF), TG, HDL, TC/HDL, ApoB, ApoA1/ApoB, Fg, pulmonary artery pressure (PAP), high GS group, TVD, and PCSK6 rs1531817 genotypes were associated with the occurrence of MACEs (Table S4). Since TC/HDL was significantly related to TG (*r* = 0.58, *P* < 0.01) and HDL (*r*=-0.65, *P* < 0.01), ApoA1/ApoB was related to ApoB, TG, HDL, and ApoB were not included in the multivariate Cox regression. After adjusting for the above statistically significant variables, multivariate Cox regression analysis revealed an independent association between stroke, TC/HDL, PAP, TVD, and PCSK6 rs1531817 genotype and the occurrence of MACEs (Fig. 4G). Among them, the CA, AA, and CA + AA genotype (adj*HR* = 0.45, 95% CI: 0.27–0.77, *P* < 0.01) were protective indicators against MACEs (Fig. 4H).

### The mediating role of lipid indicators in the association of PCSK6 rs1531817 genotypes with coronary stenosis and MACEs

**Fig. 5:**
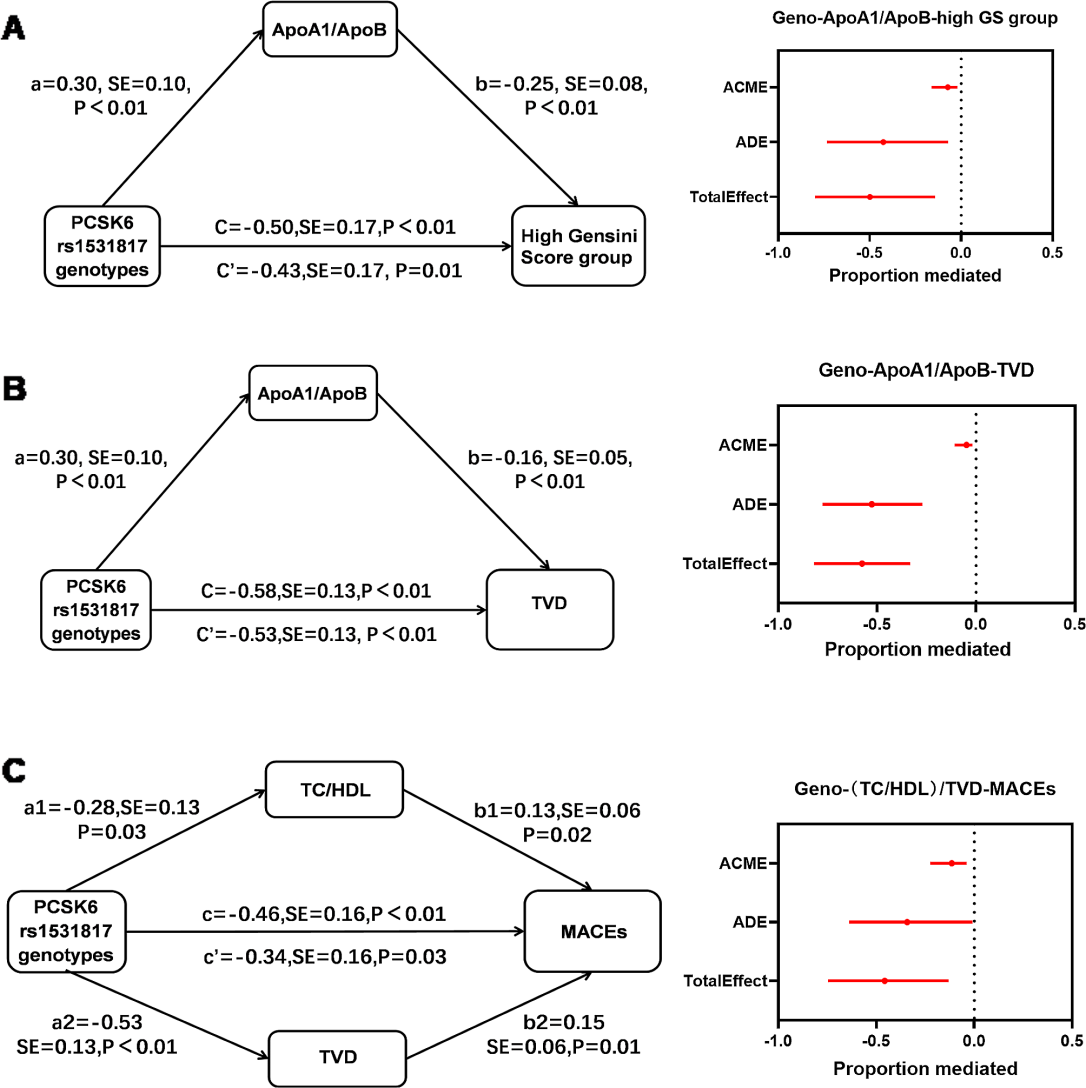
The mediating effect of lipid indicators associated with the PCSK6 rs1531817 polymorphism, coronary stenosis, and MACEs. **A** Association between the PCSK6 rs1531817 genotype and high GS mediated by ApoA1/ApoB. **B** Association between the PCSK6 rs1531817 genotype and TVD mediated by ApoA1/ApoB. **C** Association between the PCSK6 rs1531817 genotype and MACEs mediated by TC/HDL and TVD. *ADE* average direct effect; *ACME* average causal mediation effect; *SE* standard error

The above results suggested that the PCSK6 rs1531817 genotype was associated with ApoA1/ApoB, high GS and TVD (Fig. 2, Fig.S1, and Fig.S3), and that ApoA1/ApoB was associated with high GS and TVD (Fig.S1 and Fig. 3), which meets the basic requirements of mediation analysis. We used the PCSK6 rs1531817 genotypes (CA + AA vs. CC) as the independent variable, ApoA1/ApoB as the mediator variable, and high GS and TVD as the dependent variables for mediation analysis. The results showed that ApoA1/ApoB partially mediated the association between PCSK6 rs1531817 genotypes (CA + AA vs. CC) and high GS (*β*=-0.07, 95% CI: -0.16, -0.02), 14.6% of which was mediated; otherwise, we found that ApoA1/ApoB partially mediated the association between the PCSK6 rs1531817 genotypes (CA + AA vs. CC) and TVD in patients with PMI (*β*=-0.05, 95% CI: -0.11, -0.02), representing an 8.3% difference (Table S5, Fig. 5A, B).

The PCSK6 rs1531817 genotype was correlated with TC/HDL, TVD, and MACEs (Figs. 2 and 4G), and TC/HDL and TVD were associated with the occurrence of MACEs (Fig. 4G), which meets the basic requirements of parallel mediation analysis. We used the PCSK6 rs1531817 genotype (CA + AA vs. CC) as the independent variable, TC/HDL and TVD as the mediator variables, and MACEs as the dependent variable for parallel mediation analysis. The results showed that TC/HDL (*β*=-0.04, 95% CI: -0.11, -0.01) and TVD (*β*=-0.08, 95% CI: -0.17, -0.02) partially and in parallel mediated the association between the PCSK6 rs1531817 genotypes (CA + AA vs. CC), and the MACEs-mediated proportions were 8.1% and 17.1%, respectively (Table S5, Fig. 5C).

## Discussion

This prospective cohort study revealed for the first time that the PCSK6 rs1531817 polymorphism was significantly associated with severe coronary stenosis and long-term prognosis. The regression model showed that carrying the PCSK6 rs1531817 mutant A allele was an independent protective factor against severe coronary stenosis and long-term prognosis in PMI patients. To explore the potential underlying mechanisms, the study performed mediating effect analyses, which revealed that ApoA1/ApoB partially mediated the association between the PCSK6 rs1531817 polymorphism and coronary artery stenosis severity, and TC/HDL and TVD partially and in parallel mediated the association between the PCSK6 rs1531817 polymorphism and MACEs.

Endothelial lipase (EL) and lipoprotein lipase (LPL) cleavage are induced by PCSK6 in order to reduce the activity of these enzymes [25, 26]. According to previous studies, plasma ApoB expression is significantly greater in mice deficient in EL than in normal mice, suggesting that EL activity is negatively correlated with ApoB and is not correlated with ApoA [27]. EL levels have also been found to negatively correlate with serum ApoB levels in clinical studies [28]. HDL levels were elevated by LPL activation in rats given the LPL-inducing reagent (NO-1886), indicating a positive correlation between LPL activity and serum HDL concentration [29]; EL plays a crucial role in HDL catabolism by hydrolyzing phospholipids in HDL [30, 31]. The above studies suggest that PCSK6 mediates EL and LPL inactivation to regulate HDL content. In individuals with metabolic syndrome, EL serum levels and TC concentrations were found to be negatively correlated by Klobucar et al. [28]. Decreased PCSK6 expression in patients with PCSK6 rs1531817 mutations in healthy aortas (from the public GTEx resource, *n* = 197) was shown in a study by Rykaczewska et al. [10]. The differences in the ApoA1/ApoB and TC/HDL ratios among the different PCSK6 genotypes in this study were possibly due to decreased PCSK6 expression caused by the A allele of the PCSK6 rs1531817 mutation, which reduces the cleavage of LPL and EL and increases the levels of LPL and EL, it consequently results in lower TC/HDL levels and higher ApoA1/ApoB levels.

In this study, in addition to the PCSK6 rs1531817 polymorphism, the ApoA1/ApoB polymorphism was identified as an independent protective factor against TVD, TC/HDL was a risk factor for MACEs. ApoA1 reflects the sum of anti-atherosclerotic HDL particulates, and ApoB reflects the sum of atherogenic lipid particulates. In a prospective cohort study, the relation of lipid indices to MI was analyzed in 40,430 patients with atherosclerosis treated with statins at 2.5 years follow-up and in 389,529 patients in the primary prevention group who did not receive lipid-lowering therapy at 11.1 years follow-up. The findings demonstrated that the independent factor influencing the likelihood of having lipid-associated MI was ApoB [32]. In peritoneal dialysis patients, Tianlei Chen et al. analyzed the relation of ApoA1/ApoB to acute coronary syndrome, and the results indicated that ApoA1/ApoB has critical clinical value in the progression of atherosclerotic disease [33]. Fuxue Deng et al. studied 320 atherosclerotic cardiovascular disease (ASCVD) patients treated with PCI to analyze the association between ApoB/ApoA1 and coronary vulnerable plaque; plaque rupture, erosion, and thrombosis were found to be independently predicted by the ApoB/ApoA1 ratio in individuals with ASCVD [34]. The above studies showed that ApoA1/ApoB could more accurately reflect the impact of dyslipidemia on the severity of CHD than traditional lipid indicators, which is in line with the findings of this research. The TC/HDL ratio is a new indicator that has been proposed to predict CHD in recent years. TC and HDL are representative lipid indicators that promote and inhibit atherosclerosis. Calling et al. followed 6,147 women aged 50–59 years for a median time of 17 years and found that the TC/HDL ratio was a robust predictive indicator for AMI [35]. However, TC/HDL was not found to be associated with coronary stenosis in this study. In a survey of 4,957 coronary artery disease individuals, Elshazly et al. assessed the correlation of TC/HDL and the 2-year incidence of MACEs and found that a greater TC/HDL ratio was linked to a worse prognosis [36]; this finding is consistent with this study.

Atherosclerosis is significant for myocardial infarction [37, 38]. PCSK9 serves as a danger marker for atherosclerosis. Its classical pathway regulates circulating LDL levels through degradation of the LDL receptor, the elevated plasma LDL is a risk factor for atherosclerosis [39, 40]. PCSK6, as a member of the PCSK family, has polymorphisms that are associated with atherosclerosis progression [10]. The PCSK6 rs1531817 mutation A allele was shown to be a protective allele for severe coronary stenosis in patients with PMI in this study. Mediation effect analysis showed that ApoA1/ApoB partially mediated the association of PCSK6 genotypes with high GS and TVD. The mutation at PCSK6 rs1531817 increased the ApoA1/ApoB ratio, which in turn protected against severe coronary stenosis. The PCSK6 rs1531817 A allele is a protective allele for long-term prognosis; to date, there are no previous studies in this area. The mediation effect analysis in this study showed that TC/HDL and TVD played a partially parallel mediating role in the association between the PCSK6 genotypes and the occurrence of MACEs. Mutation of PCSK6 rs1531817 decreased the TC/HDL ratio and the incidence of TVD, thereby improving the long-term prognosis.

### Study strengths and limitations

Previous studies have mostly investigated the prediction of coronary stenosis and prognostic risk in PMI patients from the clinical perspective. The present study is the first to analyse the relationships among PCSK6 polymorphisms, coronary stenosis, and prognosis in patients with PMI from a genetic point of view. This study found that PCSK6 polymorphisms may play an anti-atherosclerotic and improve prognostic role by affecting lipid levels. which is crucial for guiding the prevention of severe coronary stenosis, better prognostic assessment, and more comprehensive treatment in PMI patients.

Nevertheless, the study has some limitations. First, this prospective cohort research was conducted at a single site and needed to be further validated by multiple centres, more patients, longer follow-up periods, and even different ethnicities and geographical regions. Second, the serum PCSK6 concentration was not detected, and the effect of genotypic mutations in PMI patients on the effect of the PCSK6 concentration and concentration on coronary stenosis and prognosis was not investigated. Finally, this study revealed that the PCSK6 genotype was associated with coronary stenosis and prognosis. Mediated effect analysis revealed that lipid indices play a partial mediating role, and further exploration of the potential mechanisms underlying the effects of PCSK6 on coronary stenosis and prognosis is crucial. Additionally, to confirm whether PCSK6 may serve as an interventional target for preventing severe coronary stenosis and improving long-term prognosis, further clinical and basic studies are required.

## Conclusion

This study’s results indicate that coronary stenosis and the prognosis of PMI patients are related to the PCSK6 rs1531817 polymorphism, the presence of the PCSK6 rs1531817 mutant A allele is an independent factor associated with the coronary stenosis and prognosis. PMI patients with the PCSK6 CA + AA genotype have milder coronary stenosis, partially because they have higher ApoA1/ApoB levels; patients with the PCSK6 CA + AA genotype have a better long-term prognosis, partially because they have lower TC/HDL levels and are less likely to have TVD. These results may provide a new perspective on clinical therapeutic strategy for anti-atherosclerosis and improved prognosis in PMI patients.

### Electronic supplementary material

Below is the link to the electronic supplementary material.

Supplementary Material 1

Supplementary Material 2

Supplementary Material 3

Supplementary Material 4

Supplementary Material 5

Supplementary Material 6

## Data Availability

No datasets were generated or analysed during the current study.

## Abbreviations

PCSK: Proprotein convertase subtilisin/kexin
SNP: Single nucleotide polymorphism
PMI: Premature myocardial infarction
GS: Gensini score
MACEs: Major adverse cardiovascular events
cIMT: Carotid intima-media thickness
TLR: Target lesion revascularization
HWE: Hardy‒Weinberg equilibrium
WBC: White blood cell
ALT: Alanine trans aminase
TC: Total cholesterol
HDL: High-density lipoprotein
Apo: Apolipoprotein
cTnT: Cardiac troponin T
BNP: B-type natriuretic peptide
Fg: Fibrinogen
PAP: Pulmonary artery pressure
SVD: Single vessel disease
DVD: Double vessel disease
TVD: Triple vessel disease
LVEF: Left ventricular ejection fraction
EL: Endothelial lipase
LPL: Lipoprotein lipase
OR: Odds ratio
HR: Hazard ratio
CI: Confidence intervals
