# Supplementary figures and images for: Association of the PCSK6 rs1531817(C/A) polymorphism with the prognosis and coronary stenosis in premature myocardial infarction patients: a prospective cohort study
Source: Lipids Health Dis. 2024 Jul 22;23:220. doi: 10.1186/s12944-024-02206-w (PMC11264971; doi:10.1186/s12944-024-02206-w)

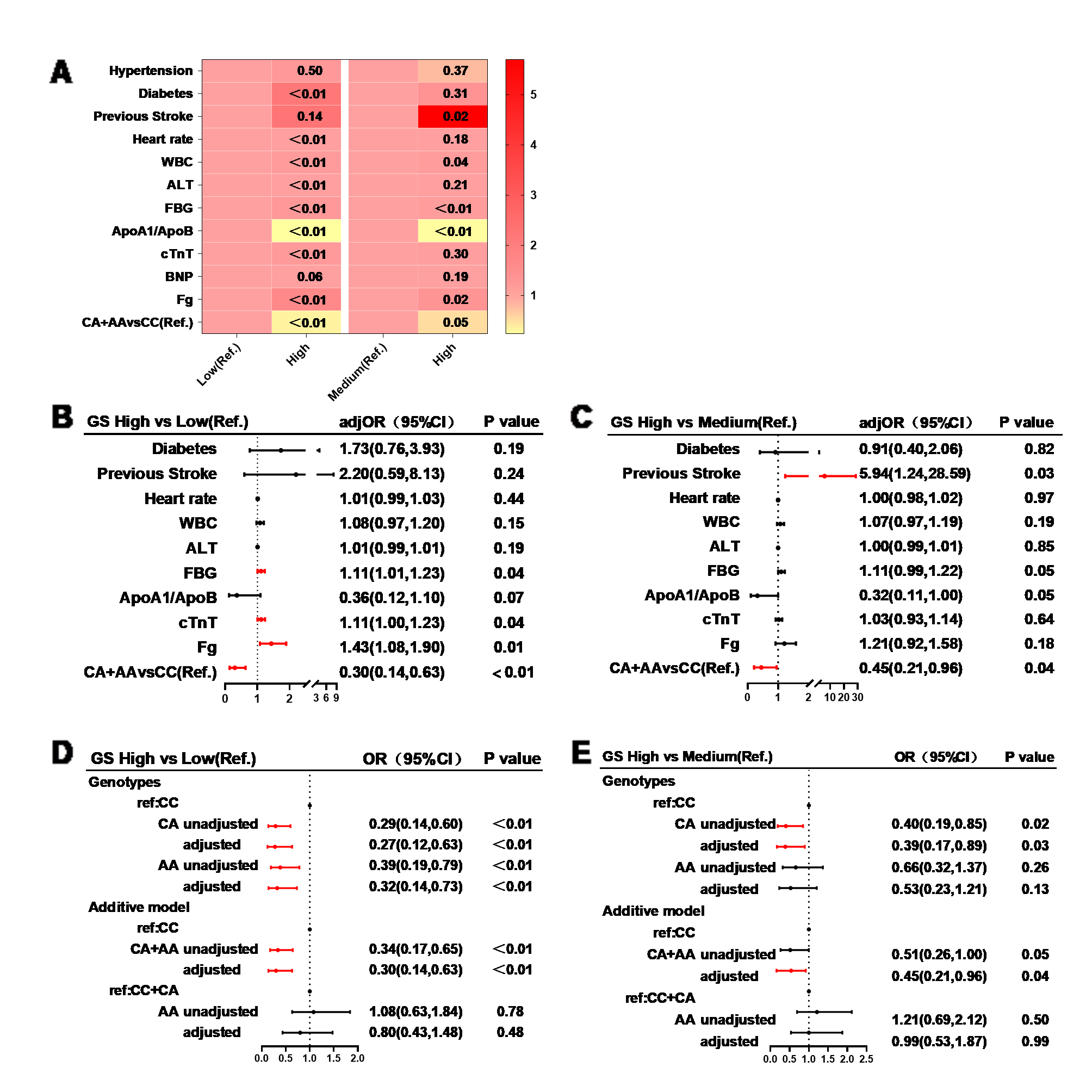

Supplement: Supplementary file 1 — Supplementary Material 1 [file 12944_2024_2206_MOESM1_ESM.tif]
