# Supplementary material for: Association of the PCSK6 rs1531817(C/A) polymorphism with the prognosis and coronary stenosis in premature myocardial infarction patients: a prospective cohort study
Source: Lipids Health Dis. 2024 Jul 22;23:220. doi: 10.1186/s12944-024-02206-w (PMC11264971; doi:10.1186/s12944-024-02206-w)
Supplement: Supplementary file 2 — Supplementary Material 2 [file 12944_2024_2206_MOESM2_ESM.docx]

**Table S1** Analysis of clinical data of PMI patients with Low GS, Medium GS and High GS

| Characteristics | Low GS group  (n=323) | Medium GS group (n=215) | High GS goup  (n=67) | *P* value |
| --- | --- | --- | --- | --- |
| Male,n(%) | 284(87.93) | 191(88.84) | 62(92.54) | 0.55 |
| Age,years | 42.00(38.00,46.00) | 42.00(38.00,45.00) | 41.00(37.00,45.00) | 0.45 |
| BMI,kg/m2 | 26.10(23.72,28.40) | 26.12(24.42,28.41) | 25.95(23.90,28.59) | 0.55 |
| History,n(%) |  |  |  |  |
| Smoking | 219(67.80) | 136(63.26) | 44(65.67) | 0.55 |
| Alcohol intake | 123(38.08) | 62(28.84) | 23(34.33) | 0.09 |
| Hypertension | 135(41.80) | 113(52.56) | 31(46.27) | 0.04 |
| Diabetes | 47(14.55) | 45(20.93) | 18(26.87) | 0.03 |
| Previous Stroke | 11(3.41) | 3(1.40) | 5(7.46) | 0.04 |
| Systolic pressure,mmHg | 132.00  (120.00,145.00) | 135.00  (123.00,145.00) | 130.00  (120.00,148.00) | 0.57 |
| Diastolic pressure,mmHg | 80.00(70.00,92.00) | 80.00(74.00,93.00) | 84.00(70.00,95.00) | 0.39 |
| Heart rate,bpm | 75.00(67.00,85.00) | 78.00(70.00,90.00) | 80.00(72.00,90.00) | ＜0.01 |
| STEMI,n(%) | 279(86.38) | 186(86.51) | 50(74.63) | 0.04 |
| Biochemical characteristics |  |  |  |  |
| WBC,10^9/L | 10.34(8.72,12.61) | 10.81(9.17,12.80) | 11.73(9.38,14.33) | 0.01 |
| CRP,mg/L | 5.33(2.14,11.40) | 5.15(2.18,11.62) | 7.08(3.08,17.61) | 0.07 |
| ALT,U/L | 42.50(29.80,64.55) | 50.40(33.50,72.80) | 62.50(31.70,88.50) | ＜0.01 |
| Cr,umol/L | 73.00(65.00,82.00) | 73.00(65.00,84.00) | 77.00(63.00,87.00) | 0.39 |
| FBG,mmol/L | 5.69(5.10,7.25) | 6.19(5.34,8.10) | 7.39(5.69,10.85) | ＜0.01 |
| TC,mmol/L | 4.72(4.10,5.38) | 4.79(4.18,5.42) | 5.07(4.36,5.82) | 0.04 |
| TG,mmol/L | 2.05(1.43,2.96) | 2.00(1.51,2.73) | 2.09(1.48,2.97) | 0.67 |
| HDL,mmol/L | 0.93(0.80,1.07) | 0.94(0.81,1.10) | 0.95(0.81,1.04) | 0.73 |
| LDL,mmol/L | 3.13(2.50,3.74) | 3.25(2.64,3.76) | 3.54(2.70,4.30) | 0.04 |
| TC/HDL | 4.97(4.18,6.24) | 5.04(4.18,5.93) | 5.39(4.59,6.69) | 0.07 |
| ApoA1,g/L | 1.13(1.01,1.26) | 1.14(1.00,1.28) | 1.13(1.05,1.26) | 0.87 |
| ApoB,g/L | 1.12(0.93,1.29) | 1.14(0.92,1.31) | 1.26(1.06,1.48) | ＜0.01 |
| ApoA1/ApoB | 1.01(0.83,1.26) | 1.01(0.84,1.19) | 0.89(0.76,1.06) | ＜0.01 |
| cTnT,ng/ml | 2.42(0.97,4.74) | 3.24(1.41,6.21) | 4.17(1.67,7.01) | ＜0.01 |
| BNP,pg/ml | 185.92  (56.81,566.13) | 253.60  (101.45,774.63) | 310.90  (69.23,970.10) | 0.04 |
| D-Dimer,ug/ml | 0.29(0.21,0.48) | 0.30(0.22,0.47) | 0.34(0.23,0.60) | 0.09 |
| Fg,g/L | 3.20(2.76,3.78) | 3.36(2.95,3.84) | 3.63(3.15,4.36) | ＜0.01 |
| PCSK6 rs1531817 C＞A |  |  |  | 0.01 |
| CC | 33(10.22） | 32(14.88） | 17(25.37） |  |
| CA | 149(46.13) | 103(47.91) | 22(32.84) |  |
| AA | 141(43.56) | 80(37.21) | 28(41.79) |  |
| Additive model |  |  |  |  |
| Dominant model(AA+CAvsCC) | 290(89.78) | 183(85.12) | 50(74.63) | ＜0.01 |
| Recessive model(AAvsCA+CC) | 141(43.65) | 80(37.21) | 28(41.79) | 0.33 |

*PCSK6* proprotein convertase subtilisin/kexin type 6; *STEMI* ST-segment elevation myocardial infarction; *BMI* body mass index; *WBC* white blood cell; *ALT* alanine transaminase; *CRP* C-reactive protein; *Cr* creatinine; *FBG* fasting blood glucose; *TC* total cholesterol; *TG* Triglyceride; *HDL* high-density lipoprotein; *LDL* low-density lipoprotein; *Apo* apolipoprotein; *cTnT* cardiac troponin T; *BNP* B type natriuretic peptide; Fg Fibrinogen.

Data are present as mean ( inter-quartile range) or number (%).
