# Supplementary material for: Association of the PCSK6 rs1531817(C/A) polymorphism with the prognosis and coronary stenosis in premature myocardial infarction patients: a prospective cohort study
Source: Lipids Health Dis. 2024 Jul 22;23:220. doi: 10.1186/s12944-024-02206-w (PMC11264971; doi:10.1186/s12944-024-02206-w)
Supplement: Supplementary file 3 — Supplementary Material 3 [file 12944_2024_2206_MOESM3_ESM.docx]

**Table S2** Analysis of clinical data of PMI individuals with SVD,DVD and TVD

| Characteristics | SVD(n=251) | DVD(n=184) | TVD（n=170） | *P* value |
| --- | --- | --- | --- | --- |
| Male,n(%) | 232(92.43) | 161(87.50) | 144(84.71) | 0.04 |
| Age,years | 42.00(37.00,44.25) | 42.50(38.00,46.00) | 43.00(39.00,46.00) | ＜0.01 |
| BMI,kg/m2 | 26.00(23.68,28.55) | 26.16(23.91,28.55) | 26.02(24.28,28.40) | 0.78 |
| History,n(%) |  |  |  |  |
| Smoking | 165(65.74) | 120(65.22) | 114(67.06) | 0.93 |
| Alcohol intake | 82(32.67) | 71(38.59) | 55(32.35) | 0.35 |
| Hypertension | 93(37.05) | 102(55.43) | 84(49.41) | ＜0.01 |
| Diabetes | 29(11.55) | 39(21.20) | 42(24.71) | ＜0.01 |
| Previous Stroke | 6(2.39) | 6(3.26) | 7(4.12) | 0.61 |
| STEMI,n(%) | 221(88.05) | 155(84.24) | 139(81.76) | 0.27 |
| Systolic pressure,mmHg | 135.00  (123.00,145.00) | 134.00  (120.25,148.00) | 130.00  (118.00,145.00) | 0.13 |
| Diastolic pressure,mmHg | 80.00(70.00,92.00) | 85.00(71.25,93.75) | 79.00(70.00,90.00) | 0.10 |
| Heart rate,bpm | 78.00(70.00,88.00) | 76.00(68.25,89.00) | 75.00(68.00,85.00) | 0.26 |
| Biochemical characteristics |  |  |  |  |
| WBC,10^9/L | 10.80(9.08,12.77) | 10.82(9.11,13.60) | 10.46(8.71,12.54) | 0.31 |
| CRP,mg/L | 5.33(2.10,11.76) | 5.71(2.74,13.74) | 5.47(2.05,11.30) | 0.47 |
| ALT,U/L | 48.20(34.40,76.95) | 46.55(30.23,69.70) | 41.85(28.85,63.40) | 0.03 |
| Cr,umol/L | 74.00(66.00,82.00) | 74.00(63.00,83.00) | 73.00(63.00,86.00) | 0.80 |
| FBG,mmol/L | 5.61(5.07,7.12) | 6.21(5.39,8.37) | 6.15(5.27,8.82) | ＜0.01 |
| TC,mmol/L | 4.65(4.07,5.24) | 4.96(4.21,5.80) | 4.87(4.20,5.63) | ＜0.01 |
| TG,mmol/L | 2.03(1.39,2.74) | 2.09(1.53,3.17) | 2.05(1.47,2.80) | 0.30 |
| HDL,mmol/L | 0.94(0.81,1.14) | 0.94(0.81,1.06) | 0.93(0.80,1.05) | 0.67 |
| LDL,mmol/L | 3.06(2.49,3.60) | 3.25(2.54,3.95) | 3.37(2.70,3.98) | ＜0.01 |
| TC/HDL | 4.79(3.91,5.98) | 5.20(4.41,6.47) | 5.27(4.36,6.09) | 0.01 |
| ApoA1,g/L | 1.14(1.02,1.29) | 1.12(1.01,1.27) | 1.12(0.99,1.25) | 0.50 |
| ApoB,g/L | 1.09(0.90,1.24) | 1.18(1.00,1.42) | 1.17(0.98,1.36) | ＜0.01 |
| ApoA1/ApoB | 1.05(0.88,1.28) | 0.99(0.78,1.18) | 0.96(0.79,1.14) | ＜0.01 |
| cTnT,ng/ml | 3.10(1.31,6.34) | 3.03(1.06,5.30) | 2.30(1.13,4.84) | 0.04 |
| BNP,pg/ml | 258.30  (76.24,709.00) | 232.79  (74.09,564.40) | 230.40  (74.00,698.60) | 0.92 |
| D-Dimer,ug/ml | 0.29(0.21,0.47) | 0.33(0.21,0.56) | 0.29(0.22,0.49) | 0.61 |
| Fg,g/L | 3.18(2.80,3.74) | 3.33(2.88,3.90) | 3.41(3.03,3.94) | ＜0.01 |
| PCSK6 rs1531817 C＞A |  |  |  | ＜0.01 |
| CC | 24(9.56) | 18(9.78) | 40(23.53) |  |
| CA | 116(46.22) | 90(48.91) | 68(40.00) |  |
| AA | 111(44.22) | 76(41.30) | 62(36.47) |  |
| Additive model |  |  |  |  |
| Dominant model(AA+CAvsCC) | 227(90.44) | 166(90.22) | 130(76.47) | ＜0.01 |
| Recessive model(AAvsCA+CC) | 111(44.22) | 76(41.30) | 62(36.47) | 0.28 |

*PCSK6* proprotein convertase subtilisin/kexin type 6; *STEMI* ST-segment elevation myocardial infarction; *BMI* body mass index; *WBC* white blood cell; *ALT* alanine transaminase; *CRP* C-reactive protein; *Cr* creatinine; *FBG* fasting blood glucose; *TC* total cholesterol; *TG* Triglyceride; *HDL* high-density lipoprotein; *LDL* low-density lipoprotein; *Apo* apolipoprotein; *cTnT* cardiac troponin T; *BNP* B type natriuretic peptide; Fg Fibrinogen; *SVD* single vessel disease; *DVD* double vessel diseas; *TVD* triple vessel diseases.

Data are present as mean ( inter-quartile range) or number (%).
