# Supplementary material for: Association of the PCSK6 rs1531817(C/A) polymorphism with the prognosis and coronary stenosis in premature myocardial infarction patients: a prospective cohort study
Source: Lipids Health Dis. 2024 Jul 22;23:220. doi: 10.1186/s12944-024-02206-w (PMC11264971; doi:10.1186/s12944-024-02206-w)
Supplement: Supplementary file 4 — Supplementary Material 4 [file 12944_2024_2206_MOESM4_ESM.docx]

**Table S3** Comparison of clinical data between all PMI patients and lost to follow-up patients

| Characteristics | All patients(n=605) | Lost to follow- up(n=56) | *P* value |
| --- | --- | --- | --- |
|  |  |  |  |
| Male,n(%) | 537(88.76) | 48(85.71) | 0.49 |
| Age,years | 42.00(38.00,45.00) | 43.00(40.00,46.00) | 0.10 |
| BMI,kg/m2 | 26.10(23.94,28.41) | 27.46(24.03,30.57) | 0.27 |
| History,n(%) |  |  |  |
| Smoking | 399(65.95) | 36(64.29) | 0.80 |
| Alcohol intake | 208(34.38) | 18(32.14) | 0.74 |
| Hypertension | 279(46.12) | 28(50.00) | 0.58 |
| Diabetes | 110(18.18) | 13(23.21) | 0.36 |
| Previous Stroke | 19(3.14) | 2(3.57) | 0.86 |
| STEMI,n(%) | 515(85.12) | 50(89.29) | 0.40 |
| Systolic pressure,mmHg | 132.00(120.00,145.00) | 130.00(121.25,144.50) | 0.84 |
| Diastolic pressure,mmHg | 80.00(70.00,92.00) | 80.00(72.75,90.00) | 0.82 |
| Heart rate,bpm | 77.00(70.00,88.00) | 77.00(69.25,90.00) | 0.72 |
| Biochemical characteristics |  |  |  |
| WBC,10^9/L | 10.73(8.94,12.81) | 11.09(9.08,13.37) | 0.40 |
| CRP,mg/L | 5.47(2.21,12.07) | 5.13(2.29,11.08) | 0.60 |
| ALT,U/L | 45.90(30.80,71.30) | 40.10(27.25,63.05) | 0.20 |
| Cr,umol/L | 74.00(65.00,83.00) | 72.00(65.00,83.75) | 0.77 |
| FBG,mmol/L | 5.94(5.22,8.01) | 6.02(5.14,7.47) | 0.70 |
| TC,mmol/L | 4.79(4.17,5.44) | 4.59(3.92,5.07) | 0.10 |
| TG,mmol/L | 2.05(1.46,2.86) | 1.85(1.38,2.93) | 0.50 |
| HDL,mmol/L | 0.94(0.81,1.08) | 0.91(0.81,1.05) | 0.55 |
| LDL,mmol/L | 3.20(2.55,3.79) | 3.06(2.54,3.56) | 0.25 |
| TC/HDL | 5.06(4.20,6.17) | 4.97(4.01,6.05) | 0.52 |
| ApoA1,g/L | 1.13(1.01,1.27) | 1.11(1.01,1.33) | 0.86 |
| ApoB,g/L | 1.14(0.93,1.32) | 1.09(0.90,1.24) | 0.16 |
| ApoA1/ApoB | 1.01(0.82,1.21) | 1.03(0.91,1.26) | 0.21 |
| cTnT,ng/ml | 2.87(1.20,5.53) | 2.70(1.26,5.02) | 0.91 |
| BNP,pg/ml | 238.05(74.66,682.05) | 182.30(45.85,667.15) | 0.37 |
| D-Dimer,ug/ml | 0.30(0.22,0.50) | 0.33(0.21,0.54) | 0.93 |
| Fg,g/L | 3.31(2.86,3.85) | 3.30(2.89,3.97) | 0.98 |
| Echocardiography |  |  |  |
| LVEF,% | 51.00(46.00,56.00) | 53.00(47.25,56.75) | 0.50 |
| PAP,mmHg | 30.00(30.00,30.00) | 30.00(30.00,30.00) | 0.99 |
| Gensini Score,points | 48.00(32.00,80.00) | 42.50(25.38,80.00) | 0.34 |
| High GS group,n(%) | 66(10.91) | 2(3.57) | 0.08 |
| TVD,n(%) | 170(28.10) | 11(19.64) | 0.18 |
| PCSK6 genotypes |  |  | 0.30 |
| CC | 82(13.55) | 6(10.71) |  |
| CA | 274(45.29) | 21(37.50) |  |
| AA | 249(41.16) | 29(51.79) |  |
| Additive model |  |  |  |
| Dominant model(AA+CAvsCC) | 523(86.45) | 50(89.29) | 0.55 |
| Recessive model(AAvsCA+CC) | 356(58.84) | 27(48.21) | 0.12 |
| Medication during follow-up,n(%) |  |  |  |
| DAPT | 602(99.50) | 56(100.00) | 0.60 |
| Statin | 595(98.35) | 55(98.21) | 0.94 |
| Anticoagulant | 547(90.41) | 48(85.71) | 0.26 |
| ACEI/ARB | 480(79.34) | 48(85.71) | 0.26 |
| Beta-blocker | 522(86.28) | 46(82.14) | 0.39 |
| HWE X^2 | 0.23 | 0.54 |  |
| HWE P | 0.63 | 0.46 |  |

*PCSK6* proprotein convertase subtilisin/kexin type 6; *STEMI* ST-segment elevation myocardial infarction; *BMI* body mass index; *WBC* white blood cell; *ALT* alanine transaminase; *CRP* C-reactive protein; *Cr* creatinine; *FBG* fasting blood glucose; *TC* total cholesterol; *TG* Triglyceride; *HDL* high-density lipoprotein; *LDL* low-density lipoprotein; *Apo* apolipoprotein; *cTnT* cardiac troponin T; *BNP* B type natriuretic peptide; Fg Fibrinogen; *LVEF* left ventricular ejection fraction; *PAP* pulmonary artery pressure; *TVD* triple vessel diseases; *DAPT* dual antiplatelet therapy; *ACEI* angiotensin-converting enzyme inhibitors; *ARB* angiotensin II receptor blockers; *MACEs* major adverse cardiovascular event; *HWE* Hardy–Weinberg law of equilibrium.

Data are present as mean ( inter-quartile range) or number (%).
