# Supplementary material for: Association of the PCSK6 rs1531817(C/A) polymorphism with the prognosis and coronary stenosis in premature myocardial infarction patients: a prospective cohort study
Source: Lipids Health Dis. 2024 Jul 22;23:220. doi: 10.1186/s12944-024-02206-w (PMC11264971; doi:10.1186/s12944-024-02206-w)
Supplement: Supplementary file 5 — Supplementary Material 5 [file 12944_2024_2206_MOESM5_ESM.docx]

**Table S4** Results of univariate Cox regression analysis of MACEs

| Characteristics | HR（95%CI） | *P* value |
| --- | --- | --- |
| Male,n(%) | 0.79(0.42,1.51) | 0.49 |
| Age,years | 0.97(0.94,1.01) | 0.10 |
| BMI,kg/m2 | 1.06(0.99,1.12) | 0.06 |
| History,n(%) |  |  |
| Smoking | 1.09(0.70,1.70) | 0.70 |
| Alcohol intake | 1.05(0.69,1.60) | 0.82 |
| Hypertension | 1.06(0.70,1.59) | 0.79 |
| Diabetes | 1.12(0.68,1.84) | 0.66 |
| Previous Stroke | 2.55(1.03,6.324) | 0.04 |
| STEMI | 1.52(0.93,2.47) | 0.10 |
| Systolic pressure,mmHg | 1.01(0.99,1.02) | 0.25 |
| Diastolic pressure,mmHg | 1.00(0.99,1.02) | 0.70 |
| Heart rate,bpm | 1.01(1.00,1.03) | 0.08 |
| Biochemical characteristics |  |  |
| WBC,10^9/L | 1.03(0.97,1.10) | 0.38 |
| CRP,mg/L | 1.00(0.99,1.01) | 0.94 |
| ALT,U/L | 0.99(0.99,1.00) | 0.26 |
| Cr,umol/L | 1.01(1.00,1.02) | 0.04 |
| FBG,mmol/L | 1.02(0.96,1.09) | 0.50 |
| TC,mmol/L | 1.17(0.98,1.40) | 0.08 |
| TG,mmol/L | 1.12(1.02,1.22) | 0.02 |
| HDL,mmol/L | 0.38(0.15,0.98) | 0.04 |
| LDL,mmol/L | 1.09(0.90,1.33) | 0.39 |
| TC/HDL | 1.16(1.06,1.28) | ＜0.01 |
| ApoA1,g/L | 0.53(0.19,1.46) | 0.22 |
| ApoB,g/L | 1.86(1.01,3.43) | 0.04 |
| ApoA1/ApoB | 0.50(0.25,0.97) | 0.04 |
| cTnT,ng/ml | 1.02(0.95,1.09) | 0.61 |
| BNP,pg/ml | 1.00(1.00,1.00) | 0.07 |
| D-Dimer,ug/ml | 0.97(0.78,1.22) | 0.82 |
| Fg,g/L | 1.21(1.02,1.43) | 0.03 |
| Echocardiography |  |  |
| LVEF,% | 0.96(0.94,0.99) | ＜0.01 |
| PAP,mmHg | 1.10(1.05,1.16) | ＜0.01 |
| Gensini Score,points | 1.01(1.00,1.01) | ＜0.01 |
| High GS group,n(%) | 2.31(1.39,3.82) | ＜0.01 |
| TVD,n(%) | 1.93(1.28,2.92) | ＜0.01 |
| PCSK6 rs1531817 C＞A |  |  |
| CC | 1 |  |
| CA | 0.41(0.24,0.70) | ＜0.01 |
| AA | 0.42(0.24,0.72) | ＜0.01 |
| Additive model |  |  |
| Dominant model(AA+CAvsCC) | 0.41(0.26,0.67) | ＜0.01 |
| Recessive model(AAvsCA+CC) | 0.79(0.52,1.21) | 0.28 |
| Medication during follow-up,n(%) |  |  |
| DAPT | 0.23(0.03,1.64) | 0.14 |
| Statin | 0.32(0.10,1.02) | 0.05 |
| Anticoagulant | 1.08(0.56,2.09) | 0.82 |
| ACEI/ARB | 0.99(0.59,1.66) | 0.98 |
| Beta-blocker | 1.00(0.55,1.84) | 0.99 |

*PCSK6* proprotein convertase subtilisin/kexin type 6; *STEMI* ST-segment elevation myocardial infarction; *BMI* body mass index; *WBC* white blood cell; *ALT* alanine transaminase; *CRP* C-reactive protein; *Cr* creatinine; *FBG* fasting blood glucose; *TC* total cholesterol; *TG* Triglyceride; *HDL* high-density lipoprotein; *LDL* low-density lipoprotein; *Apo* apolipoprotein; *cTnT* cardiac troponin T; *BNP* B type natriuretic peptide; Fg Fibrinogen; *LVEF* left ventricular ejection fraction; *PAP* pulmonary artery pressure; *TVD* triple vessel diseases; *DAPT* dual antiplatelet therapy; *ACEI* angiotensin-converting enzyme inhibitors; *ARB* angiotensin II receptor blockers.

Data are present as mean ( inter-quartile range) or number (%).
