# Supplementary material for: Association of the PCSK6 rs1531817(C/A) polymorphism with the prognosis and coronary stenosis in premature myocardial infarction patients: a prospective cohort study
Source: Lipids Health Dis. 2024 Jul 22;23:220. doi: 10.1186/s12944-024-02206-w (PMC11264971; doi:10.1186/s12944-024-02206-w)
Supplement: Supplementary file 6 — Supplementary Material 6 [file 12944_2024_2206_MOESM6_ESM.docx]

**Table S5** The associations between PCSK6 genotypes, coronary stenosis, and MACEs are mediated by lipid indicators

|  | Effect type | Effect (95%CI) | OR (95%CI) | Mediated Proportion |
| --- | --- | --- | --- | --- |
| Model 1 | TE | -0.50(-0.80,-0.14) | 0.61(0.45,0.87) |  |
|  | ADE | -0.43(-0.73,-0.07) | 0.65(0.48,0.93) | 85.2% |
|  | ACME | -0.07(-0.16,-0.02) | 0.93(0.85,0.98) | 14.6% |
| Model 2 | TE | -0.58(-0.82,-0.33) | 0.56(0.44,0.73) |  |
|  | ADE | -0.53(-0.78,-0.27) | 0.59(0.46,0.76) | 91.5% |
|  | ACME | -0.05(-0.11,-0.02) | 0.95(0.89,0.98) | 8.3% |
| Model 3 | TE | -0.46(-0.75,-0.13) | 0.63(0.47,0.88) |  |
|  | ADE | -0.34(-0.64,-0.01) | 0.710(0.53,0.99) | 74.8% |
|  | ACME | -0.12(-0.23,-0.04) | 0.89(0.80,0.96) | 25.2% |
|  | Ind1 | -0.04(-0.11,-0.01) | 0.96(0.89,0.99) | 8.1% |
|  | Ind2 | -0.08(-0.17,-0.02) | 0.94(0.84,0.98) | 17.1% |

Model 1:Geno-ApoA1/ApoB-GSgroup,

Model 2:Geno-ApoA1/ApoB-TVD,

Model 3:Geno-(TC/HDL)/TVD-GSgroup,

Ind1:PCSK6 rs1531817 genotypes-TC/HDL-MACE,

Ind2:PCSK6 rs1531817 genotypes-TVD-MACE.

*TE* total effect; *ADE* average direct effect; *ACME* average causal mediation effect; *OR* odds ratio; *CI* confidence interval.
